# Supplementary material for: The SARS-CoV-2 Alpha variant was associated with increased clinical severity of COVID-19 in Scotland: A genomics-based retrospective cohort analysis
Source: PLoS One. 2023 Apr 13;18(4):e0284187. doi: 10.1371/journal.pone.0284187 (PMC10101505; doi:10.1371/journal.pone.0284187)
Supplement: S1 Table — (DOCX) [file pone.0284187.s001.docx]

##### **Table S1: Characteristic mutations of the Alpha variant.**

| Gene | Amino acid |
| --- | --- |
| ORF1a | T1001I |
| ORF1a | A1708D |
| ORF1a | I2230T |
| ORF1a | del3675/3677 |
| ORF1b | P314L |
| S | del69/70 |
| S | del144/145 |
| S | N501Y |
| S | A570D |
| S | D614G |
| S | P681H |
| S | T716I |
| S | S982A |
| S | D1118H |
| ORF8 | Q27* |
| ORF8 | R52I |
| ORF8 | Y73C |
| N | D3L |
| N | R203K |
| N | G204R |
| N | S235F |
