# Supplementary material for: The SARS-CoV-2 Alpha variant was associated with increased clinical severity of COVID-19 in Scotland: A genomics-based retrospective cohort analysis
Source: PLoS One. 2023 Apr 13;18(4):e0284187. doi: 10.1371/journal.pone.0284187 (PMC10101505; doi:10.1371/journal.pone.0284187)
Supplement: S2 Table — (DOCX) [file pone.0284187.s002.docx]

**Table S2: Full lineage characterisation of clinical severity dataset**

| Lineage | Count |
| --- | --- |
| A.23.1 | 1 |
| B.1 | 1 |
| B.1.1.1 | 4 |
| B.1.1.10 | 1 |
| B.1.1.163 | 1 |
| B.1.1.250 | 1 |
| B.1.1.311 | 18 |
| B.1.1.315 | 6 |
| B.1.1.37 | 11 |
| B.1.1.7 (Alpha) | 364 |
| B.1.160 | 13 |
| B.1.177 | 1030 |
| B.1.2 | 1 |
| B.1.221 | 2 |
| B.1.235 | 2 |
| B.1.258 | 10 |
| B.1.351 | 3 |
| B.1.36 | 3 |
| B.1.389 | 1 |
| B.1.88 | 1 |
| P.2 | 1 |
