# Supplementary material for: The SARS-CoV-2 Alpha variant was associated with increased clinical severity of COVID-19 in Scotland: A genomics-based retrospective cohort analysis
Source: PLoS One. 2023 Apr 13;18(4):e0284187. doi: 10.1371/journal.pone.0284187 (PMC10101505; doi:10.1371/journal.pone.0284187)
Supplement: S3 Table — (DOCX) [file pone.0284187.s003.docx]

**Table S3: Parameter estimates (on the linear predictor scale) from the severity model from the full dataset**

|  | Median | Lower Bound | Upper Bound |
| --- | --- | --- | --- |
| Intercept 1 | 0.81 | 0.55 | 1.10 |
| Intercept 2 | 1.7 | 1.44 | 2.01 |
| Intercept 3 | 1.89 | 1.62 | 2.21 |
| Alpha variant | 0.34 | 0.02 | 0.66 |
| Male Sex | 0.45 | 0.23 | 0.68 |
| Linear effect of age | 1.18 | -0.34 | 3.28 |
| Linear effect of date | -0.09 | -0.81 | 0.14 |
