# Supplementary material for: The SARS-CoV-2 Alpha variant was associated with increased clinical severity of COVID-19 in Scotland: A genomics-based retrospective cohort analysis
Source: PLoS One. 2023 Apr 13;18(4):e0284187. doi: 10.1371/journal.pone.0284187 (PMC10101505; doi:10.1371/journal.pone.0284187)
Supplement: S4 Table — (DOCX) [file pone.0284187.s004.docx]

**Table S4: Parameter estimates (on the linear predictor scale) from the severity model from the data subset excluding patients in nursing homes**

|  | Median | Lower Bound | Upper Bound |
| --- | --- | --- | --- |
| Intercept 1 | 0.86 | 0.52 | 1.28 |
| Intercept 2 | 1.89 | 1.55 | 2.33 |
| Intercept 3 | 2.14 | 1.79 | 2.58 |
| Alpha variant | 0.17 | -0.21 | 0.55 |
| Male Sex | 0.50 | 0.26 | 0.75 |
| Linear effect of age | 1.16 | -0.52 | 3.40 |
| Linear effect of date | -0.03 | -0.48 | 0.08 |
