# Supplementary material for: The SARS-CoV-2 Alpha variant was associated with increased clinical severity of COVID-19 in Scotland: A genomics-based retrospective cohort analysis
Source: PLoS One. 2023 Apr 13;18(4):e0284187. doi: 10.1371/journal.pone.0284187 (PMC10101505; doi:10.1371/journal.pone.0284187)
Supplement: S5 Table — (DOCX) [file pone.0284187.s005.docx]

**Table S5: Parameter estimates (on the linear predictor scale) from the severity model from the data subset only including hospitalised patients**

|  | Median | Lower Bound | Upper Bound |
| --- | --- | --- | --- |
| Intercept 1 | -0.43 | -0.72 | -0.14 |
| Intercept 2 | 0.79 | 0.51 | 1.08 |
| Intercept 3 | 1.03 | 0.75 | 1.33 |
| Alpha variant | 0.11 | -0.28 | 0.50 |
| Male Sex | 0.28 | 0.03 | 0.52 |
| Linear effect of age | 0.84 | -0.32 | 2.84 |
| Linear effect of date | 0.02 | -0.41 | 0.42 |
