# Supplementary material for: The SARS-CoV-2 Alpha variant was associated with increased clinical severity of COVID-19 in Scotland: A genomics-based retrospective cohort analysis
Source: PLoS One. 2023 Apr 13;18(4):e0284187. doi: 10.1371/journal.pone.0284187 (PMC10101505; doi:10.1371/journal.pone.0284187)
Supplement: S6 Table — (DOCX) [file pone.0284187.s006.docx]

**Table S6: Parameter estimates from the Ct value model**

|  | Median | Lower Bound | Upper Bound |
| --- | --- | --- | --- |
| Intercept | 21.95 | 16.69 | 23.66 |
| Alpha variant | -2.46 | -4.22 | -0.70 |
| Male Sex | 0.70 | -0.78 | 2.18 |
| Linear effect of age | 0.02 | -0.56 | 1.09 |
| Linear effect of date | 0.12 | -0.57 | 1.23 |
