# Supplementary material for: The SARS-CoV-2 Alpha variant was associated with increased clinical severity of COVID-19 in Scotland: A genomics-based retrospective cohort analysis
Source: PLoS One. 2023 Apr 13;18(4):e0284187. doi: 10.1371/journal.pone.0284187 (PMC10101505; doi:10.1371/journal.pone.0284187)
Supplement: S1 Appendix — (DOCX) [file pone.0284187.s007.docx]

Appendix 1 – Further methods

Following Volz et al (2020), we modelled the ordinal severity data using cumulative generalised additive mixed models (per the definition of (per the definition of Bürkner and Vuorre (2019)) (18,20). We analysed three subsets of the data: 1. the full dataset, 2. the dataset excluding care home patients, and 3. exclusively the hospitalised population. The impact of Alpha variant infection and patient sex were modelled with fixed effects. County and partial postcode were modelled as random effects. Patient age and the days since the first diagnosis in the dataset were modelled using non-linear penalised regression splines with the *k* parameter set to its maximum value. The full dataset was additionally analysed using a phylogenetic cumulative generalised additive mixed model (PGAMM), to ensure that the results were robust to the violation of the independent errors assumption caused by the relatedness between viral isolates. The PGAMM was a modification of the GAMMs described above, where instead of including Alpha variant status as a fixed effect, we included a random effect of phylogenetic relationship between viral isolates (using a variance-covariance matrix calculated from the virus phylogeny under a Brownian motion assumption using the vcv.phylo function in ape (v. 5.5) (1)). All severity models were fitted using the brms (v. 2.14.4) R package (2). All presented models had no divergent transitions and effective sample sizes of over 200 for all parameters. Additionally, we fitted Bernoulli models with the same covariate set as the cumulative model for no oxygen vs. low flow supplemental oxygen, low flow supplemental oxygen vs. IV/NIV/HFNC and, IV/NIV/HFNC vs. mortality individually to test the proportional odds assumption.

Comorbidities were only available for patients from the Greater Glasgow and Clyde health board (n = 639). Comorbidities used were those previously identified as important for COVID-19 severity by the ISARIC4C consortium (3). To test whether the lack of comorbidity data for the rest of the sample was leading to biased estimates of the impact of Alpha variant infection, we performed three analyses on the Greater Glasgow and Clyde patient population. We fit the above model with the number of comorbidities a patient exhibited included as non-linear penalised regression spline. While the exact form of the relationship between severity of infection and the number of comorbidities a patient exhibits is unknown, we would expect the relationship to be monotonically increasing, however, for mathematical simplicity, we do not enforce this constraint on the spline. We also fit the model to this patient population without the comorbidities included and with the comorbidities permuted to estimate the change in the estimate of the Alpha variant effect by the inclusion of comorbidities. As the inclusion of comorbidities was found not to change the estimated effect of the Alpha variant, this analysis is presented in Supplementary Appendix 3.

Model intercepts were given t-distribution (location = 0, scale = 2.5, df = 3) priors, fixed effects were given normal (mean = 0, standard deviation = 2.5) priors, random effects and spline standard deviations were given exponential (mean = 2.5) priors.

1. Bürkner P-C. Brms: An R Package for Bayesian Multilevel Models using Stan. Journal of Statistical Software 2017;80:1-28.

2. Paradis E, Schliep K. ape 5.0: an environment for modern phylogenetics and evolutionary analyses in R. Bioinformatics 2019;35(3):526–528.

3. Knight SR, Ho A, Buchan I, et al. Risk stratification of patients admitted to hospital with covid-19 using the ISARIC WHO Clinical Characterisation Protocol: development and validation of the 4C Mortality Score. BMJ 2020;370:m3339.
