# Supplementary material for: The SARS-CoV-2 Alpha variant was associated with increased clinical severity of COVID-19 in Scotland: A genomics-based retrospective cohort analysis
Source: PLoS One. 2023 Apr 13;18(4):e0284187. doi: 10.1371/journal.pone.0284187 (PMC10101505; doi:10.1371/journal.pone.0284187)
Supplement: S2 Appendix — (DOCX) [file pone.0284187.s008.docx]

Appendix 2 – Phylogenetic severity model

The estimates of the severity per isolate shown in Fig 3 were generated by a model making several assumptions which may not be familiar to readers. The key assumptions used, and their impacts will be discussed in this appendix (see 1 for deeper discussion of some the issues involved).

The first major assumption is that the source phylogeny is known without error. This can be practically broken into two assumptions. Firstly, that tree-like evolution is the correct description of the underlying evolutionary process, i.e., that horizonal gene transfer is unimportant. This is a relatively safe assumption in SARS-CoV-2. Secondly, that the phylogenetic tree is correctly estimated. This is likely to be violated as there may be error in both the discrete branching structure (or topology) and real-valued branch lengths. While the topology may be correctly estimated, the probability of estimating all the branch lengths correctly is vanishingly small. This is unlikely to be a large practical issue however, as small errors in the branch lengths of the phylogeny are unlikely to have large impacts relative to other model misspecification issues present in all statistical analyses.

If we are willing to assume that the estimated phylogeny is good enough for our purposes, we then must assume some model of the evolution of the trait of interest across that phylogeny. This model of the change in the trait (in this case, severity) across the phylogeny is what allows the conversion of the phylogenetic tree into a variance-covariance matrix. This describes the expected covariances (rescaled to correlations) between the severities associated with infection with different genetic variants. Here we made a common simple choice and assumed Brownian motion evolution of the trait across the phylogeny. However, this model has been acknowledged as often suboptimal since its inception (1), and we can consider it particularly so here. The number of observed changes across SARS-CoV-2 genomes are relatively few, and the number of amino acid changes even fewer, with some mutations occurring repeatedly in different lineages. Few mutations with combined with semi-frequent homoplasy represent a particularly problematic case for this model, as severity would be expected to change discretely with mutations and in consistent directions when convergent changes occur (in the absence of extreme epistatic effects on severity), two things that simple Brownian motion does not allow. Future work will explore more realistic evolutionary models for change in severity with genomes, such as matching criteria, which will reduce the error potentially imposed by this assumption.

Despite the violation of some of the above assumptions, as noted, the answer generated was consistent with the non-phylogenetic method, implying robustness to the violation of the independent errors assumption used in the main analyses, and the output is illustrative, so the results are included in the main text, though not stressed.

1. Felsenstein J. Phylogenies and the comparative method. American Naturalist 1985;125(1):1-15.
