# Supplementary material for: The SARS-CoV-2 Alpha variant was associated with increased clinical severity of COVID-19 in Scotland: A genomics-based retrospective cohort analysis
Source: PLoS One. 2023 Apr 13;18(4):e0284187. doi: 10.1371/journal.pone.0284187 (PMC10101505; doi:10.1371/journal.pone.0284187)
Supplement: S3 Appendix — (DOCX) [file pone.0284187.s009.docx]

Appendix 3 - Comorbidities

In the Greater Glasgow and Clyde population for which comorbidity data was available, the model without inclusion of comorbidities estimated the odds ratio for the impact of the Alpha variant on severity as 1.06 (95% CI: 0.70, 1.58). When number of relevant comorbidities a patient had were included but permuted, to break any relationship with the response, a similar odds ratio was estimated (1.06: 95% CI: 0.70, 1.60). The inclusion of the number of relevant comorbidities a patient exhibited did not substantially change this result (odds ratio for impact of the Alpha variant: 1.13; 95% CI: 0.73, 1.72). This is not unexpected, as the distribution of comorbidities was similar between those patients infected with the Alpha variant and those infected with pre-Alpha lineage viruses.
