# Supplementary material for: The SARS-CoV-2 Alpha variant was associated with increased clinical severity of COVID-19 in Scotland: A genomics-based retrospective cohort analysis
Source: PLoS One. 2023 Apr 13;18(4):e0284187. doi: 10.1371/journal.pone.0284187 (PMC10101505; doi:10.1371/journal.pone.0284187)
Supplement: Attachment — Submitted filename: Response to Reviewers 20230216.docx [file pone.0284187.s010.docx]

**Response to Reviewers**

**PONE-D-22-22604**

**The SARS-CoV-2 Alpha variant is associated with increased clinical severity of COVID-19 in Scotland: a genomics-based retrospective cohort analysis**

Journal requirements:

When submitting your revision, we need you to address these additional requirements.

1. Please ensure that your manuscript meets PLOS ONE's style requirements, including those for file naming. The PLOS ONE style templates can be found at

https://journals.plos.org/plosone/s/file?id=wjVg/PLOSOne_formatting_sample_main_body.pdf

And <https://journals.plos.org/plosone/s/file?id=ba62/PLOSOne_formatting_sample_title_authors_affiliations.pdf>.

This has been completed.

2. Please provide additional details regarding participant consent. In the ethics statement in the Methods and online submission information, please ensure that you have specified (1) whether consent was informed and (2) what type you obtained (for instance, written or verbal, and if verbal, how it was documented and witnessed). If your study included minors, state whether you obtained consent from parents or guardians. If the need for consent was waived by the ethics committee, please include this information.

If you are reporting a retrospective study of medical records or archived samples, please ensure that you have discussed whether all data were fully anonymized before you accessed them and/or whether the IRB or ethics committee waived the requirement for informed consent. If patients provided informed written consent to have data from their medical records used in research, please include this information

Once you have amended this/these statement(s) in the Methods section of the manuscript, please add the same text to the “Ethics Statement” field of the submission form (via “Edit Submission”).

For additional information about PLOS ONE ethical requirements for human subjects research, please refer to <http://journals.plos.org/plosone/s/submission-guidelines#loc-human-subjects-research>.

The following statement has been added to the Methods section and has been entered in the ethics statement section of the submission form:

*Residual nucleic acid extracts derived from the nose-throat swabs of SARS-CoV-2 positive individuals whose diagnostic samples were submitted to the West of Scotland Specialist Virology Centre and Edinburgh Royal Infirmary Virus laboratory and were sequenced following ethical approvals from the West of Scotland Biorepository (16/WS/0207NHS) and the Lothian Biorepository (10/S1402/33). These samples were sequenced without consent following HTA legislation on consent exemption. Use of Scottish anonymised clinical data linked to virus genomic data without informed consent was granted by the Caldicott guardian for each site and by the Scottish Public Benefit and Privacy Panel (PBPP) for Health and Social Care (2122-0130).*

3. Thank you for stating the following in the Acknowledgments/ Funding Section of your manuscript:

“COG-UK is supported by funding from the Medical Research Council (MRC) part of UK Research & Innovation (UKRI), the National Institute of Health Research (NIHR) and Genome Research Limited, operating as the Wellcome Sanger Institute. Funding was also provided by UKRI through the JUNIPER consortium (grant number MR/V038613/1). Sequencing and bioinformatics support was funded by the Medical Research Council (MRC) core award (MC UU 1201412).”

We note that you have provided funding information that is not currently declared in your Funding Statement. However, funding information should not appear in the Acknowledgments section or other areas of your manuscript. We will only publish funding information present in the Funding Statement section of the online submission form.

Please remove any funding-related text from the manuscript and let us know how you would like to update your Funding Statement. Currently, your Funding Statement reads as follows:

“COG-UK is supported by funding from the Medical Research Council (MRC) part of UK Research & Innovation (UKRI), the National Institute of Health Research (NIHR) and Genome Research Limited, operating as the Wellcome Sanger Institute. Funding was also provided by UKRI through the JUNIPER consortium (grant number MR/V038613/1). Sequencing and bioinformatics support was funded by the Medical Research Council (MRC) core award (MC UU 1201412). The funders had no role in study design, data collection and analysis, decision to publish, or preparation of the manuscript.”

Please include your amended statements within your cover letter; we will change the online submission form on your behalf.

Removed funding statement from manuscript. Please see cover letter for updated statement.

4. In your Data Availability statement, you have not specified where the minimal data set underlying the results described in your manuscript can be found. PLOS defines a study's minimal data set as the underlying data used to reach the conclusions drawn in the manuscript and any additional data required to replicate the reported study findings in their entirety. All PLOS journals require that the minimal data set be made fully available. For more information about our data policy, please see http://journals.plos.org/plosone/s/data-availability.

Upon re-submitting your revised manuscript, please upload your study’s minimal underlying data set as either Supporting Information files or to a stable, public repository and include the relevant URLs, DOIs, or accession numbers within your revised cover letter. For a list of acceptable repositories, please see http://journals.plos.org/plosone/s/data-availability#loc-recommended-repositories. Any potentially identifying patient information must be fully anonymized.

Important: If there are ethical or legal restrictions to sharing your data publicly, please explain these restrictions in detail. Please see our guidelines for more information on what we consider unacceptable restrictions to publicly sharing data: http://journals.plos.org/plosone/s/data-availability#loc-unacceptable-data-access-restrictions. Note that it is not acceptable for the authors to be the sole named individuals responsible for ensuring data access.

We will update your Data Availability statement to reflect the information you provide in your cover letter.

Please see cover letter

5. One of the noted authors is a group or consortium [The COVID-19 Genomics UK (COG-UK) consortium]. In addition to naming the author group, please list the individual authors and affiliations within this group in the acknowledgments section of your manuscript. Please also indicate clearly a lead author for this group along with a contact email address.

This has been updated

Please review your reference list to ensure that it is complete and correct. If you have cited papers that have been retracted, please include the rationale for doing so in the manuscript text, or remove these references and replace them with relevant current references. Any changes to the reference list should be mentioned in the rebuttal letter that accompanies your revised manuscript. If you need to cite a retracted article, indicate the article’s retracted status in the References list and also include a citation and full reference for the retraction notice.

This has been checked and updated

Additional Editor Comments:

Dear authors

Your manuscript [ID Number PONE-D-22-22604] has passed through the review stage and is ‎ready for revision. ‎

Editorial comments

To ensure the Editor and Reviewers can recommend that your revised manuscript is ‎accepted, ‎please pay careful attention to each of the comments posted underneath ‎this email. This way we ‎can avoid future rounds of clarifications and revisions, moving swiftly to ‎a decision.‎

1. Please provide a point-by-point response to the Editor and reviewer's comments

‎2. Please highlight all the amends on your manuscript with yellow color

‎3. Use line numbering and page number in the next submission‎

This has been completed.

Other shortcomings

Considering the following statement in the method section: "Discharge status was followed up until 15th April 2021 for the hospital stay analysis.", Given the relatively long period of follow-up/observation, how did you control the possible impact of subsequent COVID-19 infections which may have occurred with different VOCs (other than the initial contracted infection) in the cases that were included early in the study (November 2020)?

The Delta variant was first detected by sequencing in Scotland in the week of the 14^th^ May 2021 ([Coronavirus (COVID-19): state of the epidemic - 14 May 2021 - gov.scot (www.gov.scot)).](https://www.gov.scot/publications/coronavirus-covid-19-state-epidemic-14-2021/) It is therefore very unlikely that the cases of Alpha infection included in our study would have been subsequently infected with Delta whilst a hospital inpatient and prior to our cut-off of 15^th^ April 2021. Alpha reinfection was rare and other variants circulating during our study period were doing so at very low numbers and therefore very unlikely to have impacted our results.

"Nosocomial COVID-19 was defined as a first positive PCR occurring greater than 48 hours following admission to hospital.", How did you exclude Nosocomial infections? add more detail.

We have added the clause *“individuals meeting this criterion were excluded from the study*” at the end of the sentence to make clear that detected probable nosocomial infections were simply deleted from the dataset.

Regarding the "Clinical severity analysis" section, with the information provided, the readers will be expected to reach a better conclusion based on the models applied in the subgroup analysis. However, confusion remains, about whether another subgroup could be applied to the model?

If, by this, you mean that other potential subgroups could have been investigated, this is true; Reviewer 1 notes healthcare workers as one possibility. However, we feel that the data were not complete enough for subgroup analysis for anything else that we had not already adjusted for in the original models, as such, we have not generated any additional subgroup analyses at this stage.

The following statement seems incomplete: "Bernoulli models looking at sequential severity categories provided weak evidence that the proportional odds assumption of the cumulative logistic model was violated. The odds ratios for the no oxygen versus low flow oxygen, and low flow oxygen versus [high flow oxygen???] were ..."

Thank you for pointing this out. The phrase *IV/NIV/HFNO* has been added.

You need to specify "worse outcomes" and "better outcomes" in the following statement (in this section and in the method): "A similar but more extreme effect was observed for the effect of biological sex, with male sex being associated with worse outcomes for the first two sequential category models (posterior median odds ratio for no oxygen versus low flow oxygen: 1.32, CI: 0.96-1.80; posterior median odds ratio for low flow oxygen versus high flow oxygen: 3.10, CI: 1.37-7.08), but better outcomes for the last (posterior odds ratio: 0.62, CI: 0.19-099)."

We have updated this to read ‘more severe outcomes’ and ‘less severe outcomes’.

For the following statements remove the parentheses of confidence intervals: "posterior median Ct change: -2.46, 95% CI: (-4.22)-(-0.70)"; and "age and sex (HR: -0.02; 95% CI: (-0.23)-0.20; p = 0.89)."

Thank you for highlighting this. The parentheses have been removed.

Due to the predominance of other "variants of concern" after the initial submission, the manuscript needs essential revision and update. Also, considering this fact, in order not to mislead the readers, the manuscript title should be corrected and the non-alpha types should be declared. It is better to add more information regarding study conducting time.

We have updated the manuscript to make it clear that this analysis compared severity of infections between Alpha and pre-Alpha variants, and to reflect on these results in the current context with regards to SARS-CoV-2 variants.

Reviewers' comments:

5. Review Comments to the Author

Please use the space provided to explain your answers to the questions above. You may also include additional comments for the author, including concerns about dual publication, research ethics, or publication ethics. (Please upload your review as an attachment if it exceeds 20,000 characters)

Reviewer #1: This is a really well written and well put together manuscript that addresses an important clinical area. I just have a couple of point which the authors could consider including either in their discussion or the methods in order to help thinking across organisations.

The spike gene drop out caused issues and delayed detection in some areas of the UK, was Scotland impacted by this and if so could the delay to diagnosis have played a role in increased disease severity?

S-gene target failure was used as a proxy method for detecting the Alpha variant in Scotland. As such no delay in diagnosis of SARS-CoV-2 infection was to be expected in comparison to pre-Alpha variants.

The analysis does not take into account any immunosuppression or underlying conditions. Have the authors therefore assumed that the variants would have behaved in the same way across clinical groups and that they would have all received the same management options?

A sub-analysis of patients for which co-morbidity data, including immunosuppression, were available, was carried out as detailed on line 240 and in Appendices 1 & 3.

The authors stated that excluded nosocomial cases but it would be useful to know if they also excluded care home outbreaks in the analysis that include these patients as management is likely to have been different.

Care homes were not excluded, but we performed a sub-analysis of just the patients in care homes and similar results were obtained.

Healthcare worker cases appear to have been handled in the same analysis as others, would it have been worth undertaking a healthcare worker sub-analysis as they may have been exposed to multiple variants, higher loading doses and have been captured differently due to healthcare working testing provision?

We agree that healthcare workers are likely to have had different infection histories to the general public and that this is likely to impact the severity of any infections, however, the healthcare worker data column in the dataset unfortunately had a high level of incompleteness (as well as fully excluding care home staff). As such, we do not believe it would be appropriate to perform a healthcare worker sub-analysis with this dataset. We have added the following sentence to the limitations:

*The effect of working in the healthcare sector on severity, driven by systematically different exposures faced by frontline caregivers, could not be adjusted for, due to incompleteness of the data regarding this variable.*

Reviewer #2: The authors are to be commended for a well-conducted study. Some minor notes follow.

-Obviously, the alpha variant has all but disappeared and is primarily of historical note. The authors acknowledge this and suggest that this work may help in setting a baseline to which future variants may be compared. This is a reasonable suggestion, but if this is to be the case, absolute measures of risk including cumulative incidence estimates may be more helpful than relative measures. The authors conducted sound inferential work to determine measures of disease severity relative to previous (i.e., non-B.1.1.7) strains, but any absolute measures are difficult to find, and the reader is left scraping bits from Table 1 and Figure 2. I suggest foregrounding some absolute measures in the results and discussion to better serve the purposes suggested.

Absolute measures of severity are difficult in this situation, given the large impact that demographic characteristics have on severity. It would be required to have a demographic reference class for severity to be measured against (e.g. a given age, sex and location), as the probability of each severity outcome is not linear in these effects. This dramatically reduces of the utility of such an absolute measure. There are methods we are aware of that could estimate the expected risk difference averaged over the population for an observational study such as this, but they require access to demographic data for the population that we do not have available. As such, we have attempted to foreground the proportion of individuals experiencing each severity outcome for Alpha and pre-Alpha in the main text, but we do not claim that these represent real absolute risks, given the dependence on the population structure of our study. As such, we have added the following text (in italics) to the results section:

Within the clinical severity cohort there were 364 Alpha cases, 1030 B.1.177 cases, and 81 cases due to one of 19 other pre-Alpha lineages (Fig 2), *of which 185 Alpha cases (51%) and 440 non-Alpha cases (38%) received oxygen or died*.

-Some of the typical potential confounding variables that spring to mind in a study like this-- age, calendar time (because of either changes in clinical care protocols or surges in hospital admissions), comorbidities-- were included as covariates in the primary model, as outlined in the supplementary material. I suggest discussing them directly, either in the Methods or Discussion, in order to forestall reader uncertainty.

Thank you for this suggestion. We have explicitly called out what we adjusted for in the Discussion and added a link to the supplementary appendix containing the comorbidities analysis in the results.

*Comorbidity data were not available for the full dataset, a sub-analysis on those cases where it could be linked indicated that comorbidities did not substantially affect relative severity estimates (Supplementary Appendix 3)*

In this analysis of hospitalised and community patients with Alpha variant and pre-Alpha variant SARS-CoV-2 infection, carried out as the Alpha variant became dominant in Scotland, we provide evidence of increased clinical severity associated with this variant at this time, *after adjusting for age, sex, geography and calendar time, as well as testing for sensitivity to number of comorbidities*.

-I wonder if the # of days since first diagnosis is an adequate control for potential hospital surges; there may have been variation in the timing of local outbreaks in the catchment area that left uncontrolled confounding.

This is true. Unfortunately, our dataset is not large enough to fit splines for each geographic location separately, which would have allowed us to adjust for this effect. We have noted this as a limitation in the Discussion, adding the clause in italics to the following sentence:

Finally, the cumulative model used *and usage of a single (not location varying) spine for the effect of time* in this analysis assumes a homogenous application of therapeutic intervention across the population.

-I believe the Fine and Gray method retains in the risk set those individuals who experienced a competing event not of interest (e.g., death, in the study of hospital stay length). Was the alpha variant associated with increased odds of death in this analysis?

A competing risks approach is not appropriate to answer this question, as once death is the outcome of interest, there are no competing risks - just censoring, as leaving hospital does not stop you from experiencing death. As such, we judge that the estimate from this model should not be used for that purpose and have not reported it.

-About 20% of samples were excluded because they could not be linked to patient records; is there a conceivable way that these could these have systematically differed from included samples by strain and outcomes (e.g., if they tended to be earlier samples among a low-income population that tends to have poorer health outcomes)?

The ~20% of samples not linked to patient records in our study were from health boards within Scotland where we did not have permission to access clinical data. We have updated the text to explain this.

Core demographic data (age, sex, partial postcode) were collected via linkage to electronic patient records *at the 7 of 14 scottish health boards (covering 78% of the scottish population) for which we had clinical data access approval,*

Reviewer #3: Thanks so much for this submission and for conducting this important research. I greatly appreciate learning more about how genomic surveillance in Scotland is conducted, gaining insight on clinical severity of confirmed cases of the alpha variant, and your rationale on why it is important to study variants that are no longer dominant. With some minor revisions, I think this paper will contribute to both PLOS and existing science on COVID-19. Please see my suggested revisions by section below:

Introduction

• Optional: “The Alpha variant, the first variant of concern (VOC), was estimated to be 50-100% more transmissible than others present," would say other lineages rather than others present

We have made this change.

• “A clear understanding of the behaviour of the Alpha variant, which emerged as a dominant variant, is needed as a baseline to compare the clinical phenotype of later variants, such as the currently dominant Omicron sub-variant BA.5.” Please cite a source stating dominant variant and verify that it is not BQ.1/BQ.1.1 or another variant when re-submitting since this is rapidly changing

This has been updated to read *‘A clear understanding of the behaviour of the Alpha variant, which emerged as a dominant variant in Scotland in the winter of 2020/21, is needed as a baseline to compare the clinical phenotype of variants of concern that have subsequently emerged. Post-Alpha variants, such as Omicron (B.1.1.529), have been shown to be able to evade vaccine-induced immunity and therefore have the potential to spread even in immunised populations (19), so a historical understanding of severity remains important, as it seems unlikely that SARS-CoV-2 infections will be brought under control in the near future.’*

Results

• Optional: For “At the time of the data used in this analysis” add a comma after this statement, consider re-wording to “at the time of data collection,”

This has been changed as suggested.

• In table 1, is it possible to make “65.6±20. 6” and “67.2±20. 8” fit on one line? Right now, the 6 and 8 look like they are the range.

This has been updated.

• For the other column in table 1, consider putting non-alpha in parentheses

This has been updated.

• How do you define community transmission in Table 1? Consider adding in-text or as a footnote as I didn't see this.

Thank you for pointing this out. We have explored this topic and it appears that different sites may have used different definitions of this variable. Community transmission was not used in any of the statistical analyses and only appeared in Table 1. As such we have used removed this row from the table.

• Optional: “1030 B.1.177 and 81 of 19 other lineage infections,” recommend rewording to say 81 cases from other lineages or something similar. I understand what you are saying, but from first glance it seemed like you were saying you had 81 of 19 lineages. Recommend to say 1030 B.1.177 cases and 81 cases of other lineage infections?

This has been reworded to say ‘Within the clinical severity cohort there were 364 Alpha cases, 1030 B.1.177 cases, and 81 cases due to one of 19 other pre-Alpha lineages.’

Discussion

• In the timeframe of this study, there was likely a very limited supply of vaccine available, particularly for healthcare workers. Were there any post-vaccination cases of COVID-19 in this study? Were there any exclusions made based on this, or is this something you don’t know? I suspect this could be a small source of bias if not accounted for. Consider mentioning vaccination status in discussion. If not knowing vaccination status is indeed a limitation, you could mention that post-vaccination cases were less likely in this time period due to the fact that most people had recently received their vaccine and the high vaccine efficacy at this time.

We do not have data on vaccination, but as you say, the number of people who’d received vaccination at is likely to have been small, as only over 75s and at risk groups were eligible at the end of the study period. We have noted this as a limitation in the discussion adding:

*Additionally, we did not have information about the vaccination status of the individuals in the study. However, our inability to adjust for this variable is not likely to have had a great impact on our conclusions, as, at the time of the study, the vaccination campaign had recently begun, with only over 75 year olds and high-risk groups eligible.*

• “In summary, the Alpha variant was found to be associated with a rapid increase in SARS-CoV-2 cases”. Would say cases of COVID-19 instead of cases of SARS-CoV-2, since COVID-19 is the disease. If using SARS-CoV-2, SARS-CoV-2 is the virus that causes COVID-19.

We have made this change.
